# Japanese and Canadian Children’s Beliefs about Child and Adult Knowledge: A Case for Developmental Equifinality?

**DOI:** 10.1371/journal.pone.0163018

**Published:** 2016-09-15

**Authors:** Stanka A. Fitneva, Elizabeth Pile Ho, Misako Hatayama

**Affiliations:** 1 Department of Psychology, Queen’s University, Kingston, Ontario K7L 3N6, Canada; 2 Department of Developmental and Clinical Studies, Miyagi Gakuin Women’s University, Sakuragaoka, Aoba-ku, Sendai 981-8557 Japan; Kyoto University, JAPAN

## Abstract

Children do not know everything that adults know, nor do adults know everything that children know. The present research examined the universality of beliefs about child and adult knowledge and their development with 4- and 7-year-old Canadian and Japanese children (*N* = 96). In both countries, all children were able to identify adult-specific knowledge and only older children displayed beliefs about child-specific knowledge. However, Japanese and Canadian children differed in whether they used their own knowledge in deciding whether a person who knew an item was a child or an adult. In addition, parental and child beliefs were related in Japan but not in Canada. These findings indicate that children growing up in different cultures may take different paths in developing beliefs about age-related knowledge. Implications for theories of socio-cognitive development and learning are discussed.

## Introduction

Theory of mind—the understanding of mental states such as beliefs and knowledge—is essential for children’s socio-cognitive functioning. Much research in this area has elucidated children’s ability to use a person’s behavior and statements to make inferences about the person’s knowledge [1–3]. Less is known about children’s representation of knowledge in relation to social group membership. Knowledge is often part of social category representations because of assumed training, responsibilities, or location of group members. And, as people can be readily categorized by age, gender, race, and occupation, group-related beliefs may have an immediate influence on children's behavior.

The present research aimed to contribute to understanding the development of children’s representation of the knowledge characterizing different groups. Specifically, we examined children’s beliefs about a person’s knowledge in relation to that person’s age in two cultural contexts: Canada and Japan. Age is perhaps the earliest dimension along which children organize their social world [4] and infants show sensitivity to people’s age by seven months [5,6]. Age-defined categories (e.g., child, adult) are also salient, universal, and have persistent influence over the lifespan [7–9].

In addition, the research probed for the existence of diverse developmental pathways in the construction of representations of others’ knowledge. Most research on theory of mind development has been looking for necessary or sufficient factors which, coupled with potential biological underpinnings, form a universal developmental pathway of development [10–13]. Alternatively, as a critical developmental attainment, understanding of the mind may be overdetermined and achievable via different mechanisms, i.e., mechanisms that vary in terms of the individual importance and integration of developmental factors [14]. Such developmental equifinality is quite common in open systems like the human organism [15,16]. The inclusion of Canadian and Japanese children afforded a preliminary look at this issue by examining the developmental correlates of children's beliefs. Cultural beliefs and values define the qualities and skills that children need to develop and shape the socialization and learning processes through which these developmental goals are achieved [17–20].

### What do Children and Adults Know?

Given the importance of the intergenerational processes of knowledge transmission, there is a long history of theorizing about children’s beliefs about knowledge in relation to age. While young children recognize cognitive differences among adults [21–23], a number of findings lend support to Piaget’s claim that children see adults as more knowledgeable than themselves [24] (see also [25]). For instance, it is only between five and seven years of age that children begin to recognize that they, not adults (in particular parents and teachers), know best their own minds [26]. In addition, preschoolers trust adults more than peers in suggestibility paradigms [27], believe that adults have greater capacity for acquiring knowledge [28], refer to them more often as sources of conventional and normative knowledge [29,30], and are more likely to faithfully imitate novel actions demonstrated by adults [31,32]. By age four, children also believe that some knowledge is *adult-specific*: they distinguish between knowledge that adults are more likely to possess than children, e.g., the meaning of “ambiguous”, and knowledge that both children and adults may possess, e.g., the meaning of “nice” [33–35].

It is less clear when children come to believe that some knowledge is *child-specific*, i.e., more typical of children than of adults. VanderBorght and Jaswal showed that preschoolers are more likely to ask a child than an adult about toys [35]. Two studies reported by Fitneva using a larger set of items and different methodologies question the generality of preschoolers’ beliefs about the existence of child-specific knowledge [33]. In both studies, 4-year-olds exhibited beliefs that adults know things that children do not but only 6-year-olds exhibited beliefs that some knowledge is more typical of children than of adults. Thus, 4-year-olds’ understanding of child-specific knowledge appears to be limited and to solidify a few years later.

The prolonged development of beliefs about child-specific knowledge is consistent with the assumption that beliefs about child and adult knowledge grow from children’s observations of child and adult behavior [33,35]. It is only with age, and the growth of their skills and independence, that children begin to encounter adults who are not caregivers and familiar with their everyday activities and environment. Other factors may also affect the development of children’s beliefs about child-specific knowledge. Children are exposed to explicit and sometimes contradictory information from parents and other adults in the form of aphorisms and proverbs (e.g., in English “an old man’s sayings are seldom untrue,” “the old forget, the young don’t know”) that may affect their beliefs. Children’s cognitions in a variety of domains are aligned with those of their parents [36,37].

Children may also capitalize on their own knowledge. Specifically, they may differentiate individuals and groups as they attribute the properties they have to the individual or group they see as more similar to themselves. By age three, they already identify themselves as children [7]. Importantly, choice behavior, as when associating a property with one of two categories, is strongly associated with prediction-based learning [38,39]. As choice involves contrast between concepts, it is conducive to developing beliefs about differences between the concepts, such as child- or adult-specific knowledge. Fitneva found a positive relation between 4-year-olds’ but not 6-year-olds’ self-reported knowledge and their decisions about whether to ask a child or an adult [33]. Thus, at least young children may refer to their own knowledge when deciding whether a child or an adult knows something better. They appear to reason that the likelihood for something to be better known by children than adults is higher if they possess that knowledge than if they do not.

### Pathways through Culture

Previous research on children’s beliefs about child and adult knowledge has been conducted in the USA and Canada. These are primarily individualist cultures that foster the development of an independent concept of the self [40–42]. In such cultures individuals are encouraged to attend to the self, to appreciate the differences between themselves and others, and to assert the self [41]. While children are expected to be nice and helpful, Shweder et al. echo a widely held view that “development in the European American style is almost synonymous with individualizing and decontextualizing the self” [43], p. 755. In contrast to individualist cultures, collectivist cultures such as Japan foster the development of an interdependent concept of the self. Individuals are encouraged to attend to others and establish harmonious relations with them rather than distinguish themselves from the group and assert their autonomy [41]. There is substantial intra-culture variability in both Canada and Japan, and moreover the need for autonomy (independence) and relatedness (interdependence) co-exist at the individual level [44,45]. However, the independent—interdependent framework captures systemic cultural differences in values, relationships, and practices [18,46] that raise questions about the universality of the developmental outcomes observed in North America. It also creates an opportunity to examine the universality of the mechanisms involved in the development of beliefs about child and adult knowledge by examining the correlates of these beliefs in different cultures.

With respect to developmental outcomes, the distinctive functions of child-child and child-adult relationships (e.g., friendship vs. care) suggest that beliefs about child- and adult-specific knowledge are universal developmental achievements. In particular, given the universal dependence of children on adults, it would be surprising if children did not develop beliefs that adults know things children do not, as well as if they did not develop these beliefs earlier than beliefs that children know things that adults do not. Similarly, given the value of peer companionship, it would be surprising if children did not develop beliefs about child-specific knowledge. Such beliefs, however, “individuate” the child relative to the rest of the community and are thus more consistent with an independent than an interdependent concept of the self. Japanese culture also places stronger emphasis on respect for the elderly through practices such as ancestor veneration and a national holiday focused on the elderly, potentially further discouraging such beliefs. The hypothesis that both Canadian and Japanese children establish beliefs about child-specific knowledge thus requires empirical confirmation.

To provide a strong test of developmental outcomes, the present research compared 4-year-olds to 7-year-olds rather than 6-year-olds as done in previous studies. It was deemed that by the second year of formal schooling (age 7–8 in both Canada and Japan), children must have enough experience to warrant such beliefs. It is possible that culture influences *when* rather than or in addition to *whether* children develop beliefs about child-specific knowledge. The present study focused on the latter question and is only informative about the former in a limited way.

Do the same factors influence Canadian and Japanese children’s age-related knowledge representations? We focus on beliefs about child-specific knowledge because the longer period over which these beliefs appear to emerge allows for greater ontogenetic variability. We examined two factors mentioned in the previous section—the content of one’s own mind and parental cognitions. As noted above, children’s beliefs about their own knowledge is the only factor that has been previously examined, receiving limited support [33]. On the other hand, parental cognitions constitute a potentially powerful, proximal environmental factor [36,37] and were relatively easy to assess given the practical restrictions on the research. It is possible that beliefs about child-specific knowledge are shaped by the same factors across culture. But it is also conceivable that Canadian and Japanese children arrive at beliefs about child-specific knowledge in somewhat different ways due to cultural differences in relationships and socialization processes which shape the empirical basis of these beliefs. As illustrated below, relationships and socialization processes are organized to foster a predominantly independent concept of the self in Canadian children and a predominantly interdependent concept of the self in Japanese children.

The role of children’s beliefs about their own knowledge relates in particular to the organization and functioning of peer groups. Even though individuality and relatedness are important in both Canada and Japan, consistent with the values of individualist cultures, the emphasis for North American children falls on maintaining an autonomous self and educational practices underscore the importance of each child more so than of the group (as when every child on a team receives a trophy [43]). In contrast, consistent with the values of collectivist cultures and fostering an interdependent self concept, Japanese parents and schools emphasize to a greater extent children’s alignment with others and group harmony [47–49]. For instance, students are expected to arrange their lunch boxes in a specific way and to participate in a group physical exercise routine at the beginning of the school day [48]. There is also a strong emphasis on the development of *omoiyari* (empathy), which refers to the expectation that individuals anticipate and prioritize the needs of others [43,49–51]. As a result of this greater emphasis on aligning one’s behavior with that of peers, Japanese children may see their knowledge as representative of what peers know more readily than Canadian children. Correspondingly, we hypothesized that there would be a stronger correlation between Japanese children’s self-reported knowledge and their decisions about whether a person with that knowledge is a child or an adult.

The relation between child and parent cognitions, on the other hand, relates to parenting practices. Two possibilities emerge from the consideration of these practices. First, Keller et al. [52] suggest that body contact and warmth, which are greater in collectivist cultures, facilitate children’s adoption of norms and beliefs espoused by parents. This suggests a stronger positive correlation between the beliefs of children and parents in Japan than in Canada. The second possibility relates to the grounding of parents’ and children’s beliefs in the interactions they have with each other. North American culture places high value on verbal self-expression as a source of knowledge about people [49,51,53]. As a result, Canadian parents may develop beliefs about children’s distinctive knowledge only as children’s verbal skills improve with age. In contrast, Japanese parents (mothers in particular) support the development of an interdependent self and skills for anticipating and meeting others’ needs through modeling the relevant behaviors themselves and being attuned to the needs of the child [50]. These practices may lead Japanese parents to develop beliefs about child-specific knowledge earlier. But, as they establish adults’ epistemic authority with respect to the child [18,51,53], they may delay the development of beliefs about child-specific knowledge in children. In other words, there may be a negative correlation between parent and child beliefs about child-specific knowledge. On the surface, this prediction contradicts the concepts of ‘collectivist culture’ and ‘interdependent self’ which may be interpreted as implying that individuals expect uniformity among members of the community. However, theoretically as well as empirically, these concepts are associated with greater attention to context rather than confounding one’s own and others’ perspectives [41,54,55]. Japanese adults are indeed more sensitive to diversity of perspectives than American adults [54].

In sum, the goal of this study was to examine the development of beliefs about adult- and child-specific knowledge in two cultural contexts. Of particular interest were the questions of universality of beliefs about child-specific knowledge and how children come to construct such beliefs. We hypothesized that beliefs about adult-specific knowledge would develop before beliefs about child-specific knowledge in both cultures. We also hypothesized that beliefs about child-specific knowledge would be more strongly, positively related to children’s own knowledge and parental beliefs in Japan, organized to foster interdependence, than Canada, where independence is emphasized. An alternative hypothesis about the relation between child and parent beliefs in Japan was that they would be negatively correlated. As explained in the previous paragraph, Japanese parenting practices could lead to recognition of child-specific knowledge by parents but highlight parental epistemic authority for children.

## Method

### Participants

In Canada, 24 4-year-old children (*M =* 4 years 6 months, range 4;0–5;0) and 24 7-year-old children (*M =* 7 years 6 months, range 7;0–8;0) participated in the study. There were 10 girls and 14 boys in the younger group and 15 girls and 9 boys in the older group. Two more 4-year-olds were tested but replaced due to inattentiveness during testing. The children were recruited in Kingston, Ontario, a mid-size urban community, and were enrolled in kindergarten or 2^nd^ grade. All were of European descent.

In Japan, 24 4-year-old children (*M* = 4 years 6 months, range 4;0–5;0) and 24 7-year-old children (*M* = 7 years 9 months, range 7;4–8;3) participated in the study. An additional 4-year-old was excluded because he did not complete the study due to school activities. The children were recruited in Sendai, Northeast Japan, and were enrolled in yochien (kindergarten with academic focus similar to that in Canadian early education) or 2^nd^ grade. All were ethnically Japanese.

The parents of 24 Canadian 4-year-olds, 23 Canadian 7-year-olds, 19 Japanese 4-year-olds, and 23 Japanese 7-year-olds also participated by filling out a questionnaire. In both countries, mothers constituted over 80% of the respondents. As part of the questionnaire, parents provided additional demographic information, which is summarized in Table 1. As expected, on average Canadian children had more siblings than Japanese children and there were more adults in the homes of Japanese children than Canadian children. The samples were well matched in maternal educational background.

**Table 1:** Child Demographics.

	Canada		Japan	
	Kindergarten (n = 24)	2^nd^ grade (n = 23)	Kindergarten (n = 19)	2^nd^ grade (n = 23)
Mean number (range) of siblings at home	1.4 (0–3)	1.5 (0–4)	.8 (0–2)	1 (0–2)
Mean number (range) of adults at home	2	2	2.7 (2–5)	2.7 (1–5)
Mothers with post-secondary education	95%	88%	89%	87%

The study was approved by the General Research Ethics Board of Queen's University. Parents provided written informed consent for their and their child’s participation. All children provided verbal assent.

### Materials

The main task in the study was an identification task in which children had to decide whether a person was a child or an adult based on what the person knew. This task was modeled after the property-to-category induction task [56]. Selecting an informant between a child and an adult [33,35] and directly asking “Does an adult know…? Does a child know…?” [33,34] were deemed not well suited for capturing beliefs that potentially contradict the imperative of deference to adults in Japan.

Twelve cards were used in the identification task. On one side of each card there was a statement and on the other a name. Half of the names were male and half female. Six statements tapped adults’ domain of knowledge and six tapped children’s domain of knowledge (two per topic, see S1 Appendix). Three topics were used for adult-domain items (cooking, language, and transportation) and three for child-domain items (games, songs, and children’s TV shows). By definition, adult-domain items refer to knowledge that is more typical of adults than of children and child-domain ones to knowledge that is more typical of children than of adults. Thus, we aimed for items with high cue validity, where cue validity is the conditional probability that a person belongs to the target adult or child category given the knowledge cue. Note that what matters is the difference in the spread of a given piece of knowledge among adults and children, not how common that knowledge is in absolute terms. Thus, even though only the few adults with specialized medical training know how to fix a broken arm, as likely no child knows, this represents adult-specific knowledge. We do not expect all adults and all children to have the knowledge captured respectively by the adult- and child-specific items. Also note that as adults take care of children and shape their activities (e.g., by creating TV shows), child-specific knowledge items likely have lower cue validity with respect to category membership than adult-specific knowledge items. We assume that this is an intrinsic feature of the problem that children need to solve in developing beliefs about child-specific knowledge.

As the above discussion suggests, it is more challenging to identify child-domain items. There is also the added challenge of selecting items that are appropriate for both 4- and 7-year-olds. As in previous research [33], we also wanted to use both procedural (how to) and factual knowledge items. As procedural knowledge cannot be easily verified, rather than attempting to objectively establish child-specific knowledge, we selected the child domain topics based on interviews of Canadian parents whose 4- to 7-year-old children participated in a different study. They were asked to describe what, if anything, their children knew better than they did. There was substantial thematic overlap in the responses regardless of child age and the selected topics captured this overlap. As the responses tended to be general (e.g., games, TV shows), we involved another group of parents and teachers to create the specific items. These informants had close and ample observations on 4- to 7-year-old children. Thus, we reasoned that they would be most likely to be able to generate items that meet the definition of ‘child specific’ and refer to activities that were familiar to both age cohorts in the study. We ascertained with a small group of Japanese parents and teachers that the same topics were appropriate in Japan. They also helped generate the specific items used with the Japanese children.

The instructions, questions, and the parental questionnaire were translated from English to Japanese and back to English by two different bilingual Japanese-English speakers. The process was iterated until all discrepancies between the original and back-translated materials were resolved.

### Procedure

Canadian children were tested in the lab while parents observed from a separate room. Japanese children were tested in an unoccupied room in their school. The experimenters were native speakers of English or Japanese and spent time building rapport with the participants in the waiting area or their classroom before obtaining their assent to participate in the study.

#### Identification task

The study began with the experimenter spreading the twelve cards in front of the child with names on top and saying that each card has the name of a person on one side and turning it reveals something that the person knows. Then the names were read out loud and the experimenter told the child that some of the people the cards were about were children and some adults and that the child’s task was to figure out who was a child and who was an adult. Special care was taken to make sure that Japanese children understood that there were both children and adults in the set as the honorific particle *san* was used with all Japanese names. (The honorifics “kun” and “chan” are more typical in reference to children but “san” is a default particle indicating respect and can be also used in reference to children.) Children were told that a child is a person who is about the same age as they are, a bit younger or older, and an adult is someone who is much older. To confirm their understanding, they were asked to name and describe at least one child and one adult.

On each experimental trial, the experimenter took a card, read who it was about, then turned the card over, read what the person knew, and asked whether the person was a child or an adult e.g., “This card is about Katrina. Katrina knows what the word ‘elaboration’ means. Is Katrina a child or an adult?” The order of “child” and “adult” was counterbalanced between subject for each item and within subject across items. Children were randomly assigned to one of four pseudorandom item presentations orders, which were constructed with the constraint that same-topic items did not appear next to each other. To provide an opportunity for non-verbal pointing response, a small and a large stick figures, introduced respectively as a child and an adult, were set in front of the children.

#### Own knowledge

After a five minute break, children reported their knowledge of the items used in the identification task, e.g., “*Do you know* what the word ‘elaboration’ means?” The main clause of the questions (in italics) was emphasized to make sure children focused on the main rather than the embedded question. The items were presented in a different order than in the identification task. Follow-up questions (e.g., “Okay, what do you think ‘elaboration’ means?”) were asked for both “yes” and “no” responses to discourage a yes-bias or responding “no” because the child did not want to talk. The answers to these questions were not analyzed because we were interested in children’s beliefs about what they knew and therefore we did not elicit exhaustive responses. That said, children’s responses to the questions about simple facts (e.g., what’s the name of Spongebob Squarepants’ best friend?) were consistent with their self-reported knowledge (i.e., children who said they knew, said “Patrick” and none of the ones who said they did not know did).

#### Metacognitive task

In an attempt to obtain converging evidence for the identification task, children were asked two metacognitive questions about the existence of child-specific knowledge, without reference to particular topics. As these questions explicitly challenge adult authority, however, we were unsure whether the task would be suitable for Japanese children. Indeed, the Japanese children were highly inconsistent in their responses, raising questions about the cultural validity of the task. Given our a priori concerns, we leave out the discussion of this task. See S2 Appendix for its description and results.

#### Parental beliefs

Parents filled out a questionnaire which included demographic questions as well as two questions about child-specific knowledge (in reference to the child participating in the study): “Is there anything you feel your child knows more about than you do?” and “Is there anything you feel your child can do better than you can do?” Parents were asked to list all of the examples of such items that they could think of to ensure that affirmative responses were not simply driven by the polarity of the questions.

## Results

### Identification Task

**Fig 1:**
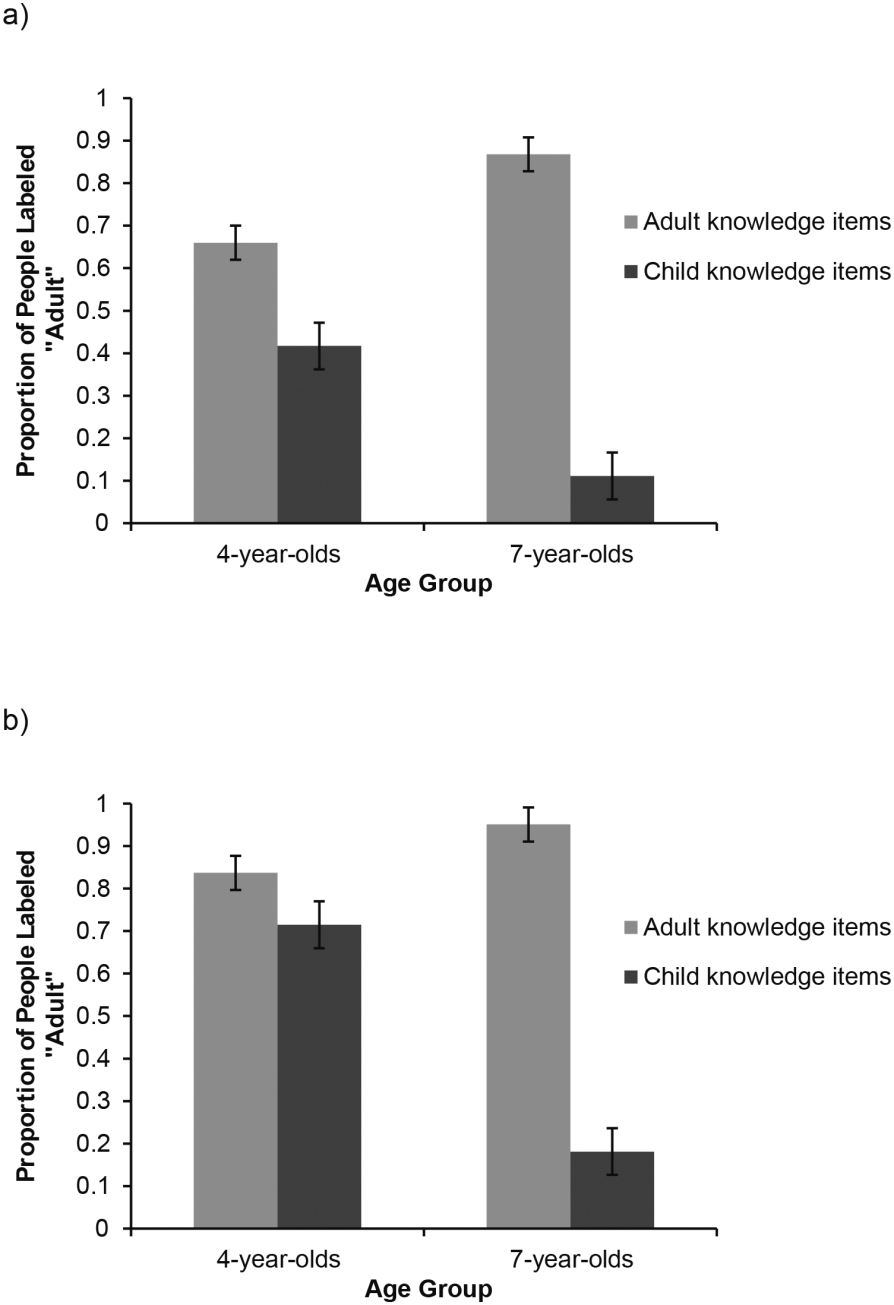
Identification Decisions as a Function of Item Domain and Children’s Age. Panel a) shows the responses of Canadian children and panel b) of Japanese children. Error bars indicate ± 1 SE.

Preliminary analyses showed no significant differences among items and topics within the adult and the child knowledge domains. Thus, the data were collapsed across the six items in each domain and the analyses were conducted on the proportion of times children identified the individuals associated with child- and adult-knowledge items as adults (Fig 1). We first examine whether and when children differentiated the two item domains. We then turn to the questions about developmental outcomes and the sequence of development of beliefs about child- and adult-specific knowledge.

#### Differentiation of knowledge domains

The data were analyzed using a repeated-measures ANOVA where the items’ domain (adult vs. child knowledge) was a within-subject variable and age (4- vs. 7-year-olds) and country (Canada vs. Japan) were between-subject variables. The ANOVA revealed a significant main effect of age, *F*(1, 92) = 9.85, *p* = .002, η_p_^2^ = .1, knowledge domain, *F*(1, 92) = 349.64, *p* < .001, η_p_^2^ = .79, and an interaction effect between knowledge domain and age, *F*(1, 92) = 132.1, *p* < .001, η_p_^2^ = .59. As Fig 1 shows, 4-year-olds were more likely than 7-year-olds to identify the characters as adults. In addition, characters possessing adult-typical knowledge were more likely to be identified as adults than characters possessing child-typical knowledge. Confirming previous findings, 7-year-olds showed greater discrimination between the adult and child domain items (*M* = .91 vs. *M* = .15, *F*(1, 46) = 495.637, *p* < .001, η_p_^2^ = .92) than 4-year-olds (*M* = .75 vs. *M* = .57, *F*(1, 46) = 24.024, *p* < .001, η_p_^2^ = .34).

The analysis also revealed a main effect of country, *F*(1, 92) = 14.54, *p* < .001, η_p_^2^ = .14, qualified by a marginal interaction effect between age and country, *F*(1, 92) = 3.84, *p* = .053, η_p_^2^ = .04. As Fig 1 suggests, Japanese children, in particular preschoolers, were more likely to identify individuals as adults compared to Canadian children. Implying similarity of the developmental trends in the two countries, the 3-way interaction between country, age, and knowledge domain was not significant, *F*(1, 92) = 1.79, *p* = .18, η_p_^2^ = .01.

We conducted further ANOVAs to more closely examine the age differences in each country and the effect of domain in each age group. The analysis of the Canadian data revealed no effect of age, *F*(1, 46) = .59, *p* = .4, η_p_^2^ = .013, a significant effect of knowledge domain, *F*(1, 46) = 150.64, *p* < .001, η_p_^2^ = .77, and an interaction between age and domain, *F*(1, 46) = 39.78, *p* < .001, η_p_^2^ = .46. Both 7-year-olds and 4-year-olds identified individuals as adults more often for adult-domain than child-domain items (*t*(23) = 14.803, *p* < .001, Cohen’s *d =* 3.887 for 7-year-olds; *t*(23) = 3.832, *p* = .001, Cohen’s *d =* .77 for 4-year-olds). The analysis of the Japanese data showed a main effect of age, *F*(1, 46) = 15.7, *p* < .001, η_p_^2^ = .26, a main effect of domain, *F*(1, 46) = 220.96, *p* < .001, η_p_^2^ = .83, and an interaction between age and domain, *F*(1, 46) = 116.99, *p* < .001, η_p_^2^ = .72. Both 7-year-olds and 4-year-olds identified individuals as adults more often for adult-domain than child-domain items (*t*(23) = 16.842, *p* < .001, Cohen’s *d =* 6.703 for 7-year-olds; *t*(23) = 3.128, *p* = .005, Cohen’s *d =* .457 for 4-year-olds). Thus, regardless of age and culture, children differentiated the child and adult knowledge items. This differentiation, however, was more pronounced in 7-year-olds than 4-year-olds, as shown by the effect sizes.

#### Developmental outcomes

To assess children’s awareness of adult- and child-specific knowledge, we compared the rate at which children identified the characters as adults or children to chance (50%). Canadian 7-year-olds’ identified the characters with adult-domain knowledge as adults 87% of the time, *t*(23) = 12.99, *p* < .001, and the characters with child-domain knowledge as children 89% of the time, *t*(23) = 7.97, *p* < .001. Canadian 4-year-olds’ identified the characters with adult-domain knowledge as adults 66% of the time, *t*(23) = 2.93, *p* = .007, and the characters with child-domain knowledge as children 58% of the time, which was not different from chance, *t*(23) = 1.163, *p* = .257.

Japanese 7-year-olds identified the characters with adult-domain knowledge as adults 95% of the time, *t*(23) = 19.22, *p* < .001, and those with child-domain knowledge as children 82% of the time, *t*(23) = 10.12, *p* < .001. Japanese 4-year-olds identified the characters with adult-domain knowledge as adults 84% of the time, *t*(23) = 7.22, *p* < .001. They identified the characters with child-domain knowledge as children only 28% of the time, which was significantly *below* chance, *t*(23) = -3.54, *p* = .002. Thus, consistent with our predictions, in Japan, as in Canada, both 4- and 7-year-olds demonstrated beliefs about adult-specific knowledge but only 7-year-olds demonstrated beliefs about child-specific knowledge. Beliefs about child-specific knowledge emerge in both cultures but beliefs about adult-specific knowledge appear to develop earlier.

### Identification and Self-Reported Knowledge

To explore the role of children’s beliefs about their own knowledge in deciding whether a child or an adult is more likely to have that knowledge, answers to the self-report questions were coded as 1 for “yes” and 0 for “no.” In a preliminary step, we explored whether children’s answers in each country varied as a function of item domain (adult vs. child) and age (4- vs. 7-year-olds). Canadian children’s responses only showed a significant effect of domain, *F*(1, 46) = 44.748, *p* < .001, η_p_^2^ = .49. As expected, children reported greater knowledge of the child-domain than the adult-domain items, 46% vs. 20%. Japanese children’s responses varied by domain as well, *F*(1, 46) = 33.261, *p* < .001, η_p_^2^ = .42, but there was also an interaction effect between domain and age, *F*(1, 46) = 11.781, *p* < .001, η_p_^2^ = .20. Self-reported knowledge of the child and adult items was 47% and 19% respectively for 7-year-olds (*t*(23) = 6.768, *p* < .001) and 35% and 28% respectively for 4-year-olds (*t*(23) = 1.592, *p* = .12).

These findings invite several observations. First, the difference between 4- and 7-year-old Japanese children invites the question whether the age differences between them in the identification task are due to differences in their knowledge. This question is addressed in the following analyses and the results suggest that this was not the case. Second, the fact that 4-year-olds claimed to know more of the adult-domain items than 7-year-olds is a reminder to not treat children’s self-reported knowledge as representing their actual knowledge. Finally, as our child participants appeared relatively unfamiliar with the child-domain items, it is important to note that this in itself does not undermine the items’ validity. Indeed, parents and teachers judged that these items were more familiar to children than adults and it is this *difference* that is key to their definition.

To examine whether children’s beliefs about their knowledge were related to their identification of a person as a child or an adult, in addition to the factors in the previous analysis (item domain, age, and country) this analysis included children’s self-reported knowledge. As each item was considered separately, the data were binary repeated measures and analyzed using generalized estimating equations (GEE). The results confirmed the major findings of the previous analysis for item domain, age, and country. Given that children’s knowledge of the items was controlled, this indicates that the age-related trends in children’s representation of child and adult knowledge were not due to developmental differences in children’s familiarity with the items.

**Fig 2:**
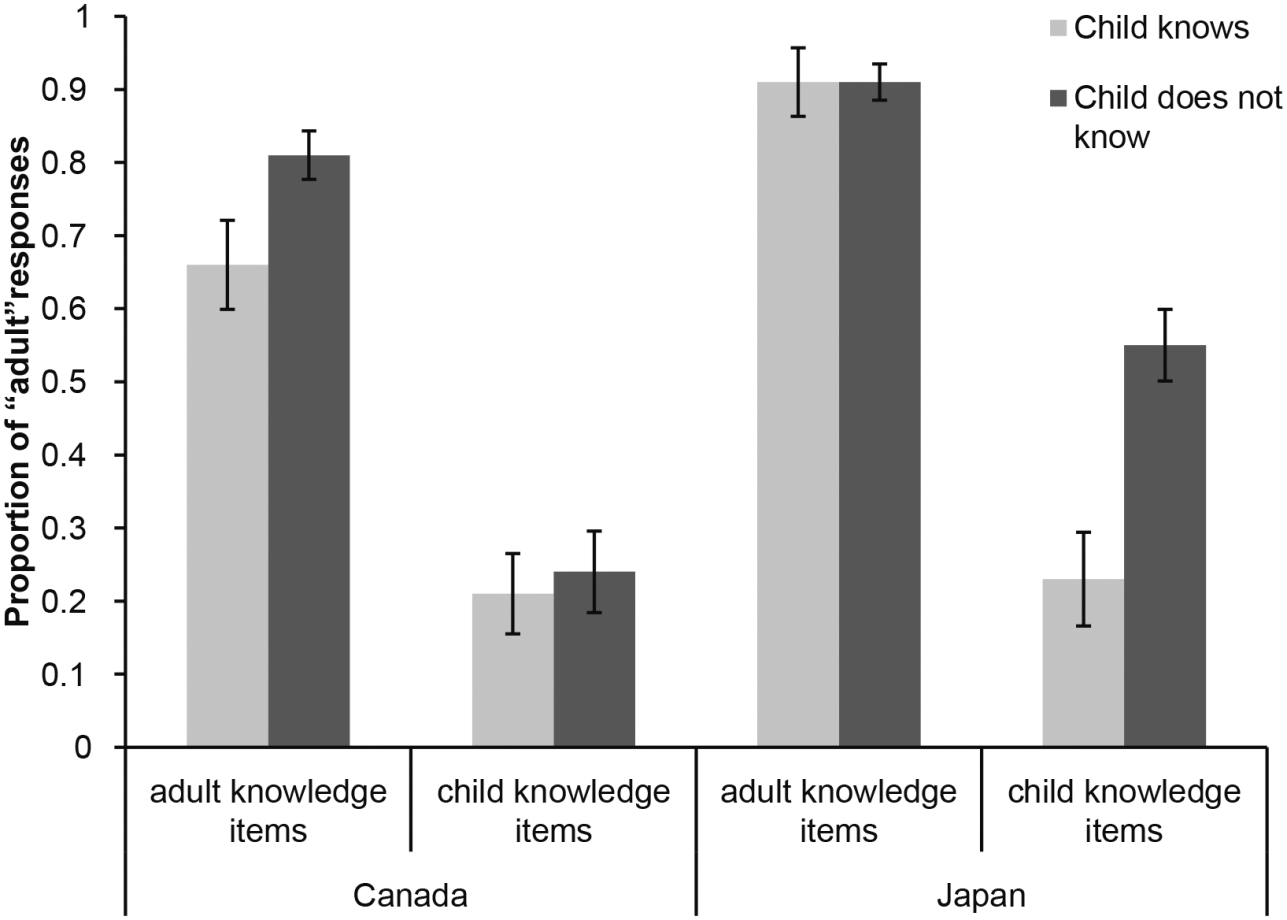
Proportion of people identified as “adult” by Canadian and Japanese children, as a function of self-reported knowledge of the items and item domain. Error bars indicate ± 1 SE.

Focusing on the effect of self-reported-knowledge, the GEE analysis revealed a significant main effect of this factor, Wald χ^2^(1) = 7.429, *p* = .006, qualified by a three-way interaction between self-reported knowledge, country, and domain, Wald χ^2^(1) = 5.933, *p* = .015. Fig 2 displays this result. Both Canadian and Japanese children were more likely to identify a person as an adult when they did not share that person’s knowledge. However, as Fig 2 suggests, Japanese children’s self reports were more strongly correlated with their responses to the child- than the adult-domain items in the identification task. No such bias was evident for Canadian children.

Follow-up analyses showed only a simple main effect of self-reported knowledge in Canada, Wald χ^2^(1) = 4.796, *p* = .029. In Japan, the effect of self-reported knowledge (Wald χ^2^(1) = 3.68, *p* = .055) was qualified by an interaction with domain, Wald χ^2^(1) = 4.07, *p* = .044. The differences in Japanese 4- and 7-year-olds’ self-reported knowledge are unlikely to account for the developmental differences in recognizing the child-domain items as such, as the 3-way interaction between self-reported knowledge, domain, and age was not significant, Wald χ^2^(1) = 2.94, *p* = .09.

As our main interest was in child-domain items, and to further explore the 3-way interaction between self-reported knowledge, domain, and country, we analyzed the data for the two item domains in each country separately. Focusing on child-domain items first, Japanese children were more likely to say that a character was an adult when they reported to not know an answer than when they reported to know it (55% vs. 23%, Wald χ^2^(1) = 14.678, *p* < .001). This trend was not significant for Canadian children, Wald χ^2^(1) = .04, *p* = .837, leading to a significant country by self-reported knowledge interaction for child-domain items, Wald χ^2^(1) = 8.096, *p* = .004. Considering adult-domain items next, the effect of self-reported knowledge was not significant in either country (Wald χ^2^(1) = .15, *p* = .7 in Japan; Wald χ^2^(1) = 1.586, *p* = .208 in Canada). The country by self-reported knowledge interaction was not significant either, Wald χ^2^(1) = .18, *p* = .688. Thus, even though in both countries children’s self-reported knowledge was related to their decisions about whether a character was a child or an adult, the relationship was strongest for Japanese children’s decisions about child-domain items.

### Parental Beliefs

Caregivers’ responses to the questions about whether their children possessed knowledge that they did not were coded as 1 for “yes” and 0 for “no.” Restricting the analyses to mothers did not affect the results. Using the responses to each question separately showed similar results and the data for the two questions were correlated (*r* = .4, *p* < .001). Consequently, the following analyses used the average of parents’ responses to the two questions.

Again, in a preliminary step, we summarize the parent responses. The proportion of affirmative parental responses was analyzed as a function of child age (4 vs. 7) and country. The analysis revealed a significant effect of child age, *F*(1, 84) = 4.69, *p* = .03, η_p_^2^ = .053, country, *F*(1, 84) = 12.687, *p* = .001, η_p_^2^ = .13, and an interaction between age and country, *F*(1, 84) = 4.194, *p* = .044, η_p_^2^ = .048. Canadian parents reported that their children know things that they do not 61% of the time. Parents of 7-year-olds were significantly more likely to do so than parents of 4-year-olds: 76% vs. 46%, *F*(1, 45) = 7.567, *p* = .009, η_p_^2^ = .14. Japanese parents reported child-specific knowledge 86.5% of the time, and there was no effect of child age, 87% vs. 86%, *F*(1, 40) < 1.

How do parents’ beliefs about child-specific knowledge relate to their children’s beliefs? To examine this relation, we regressed the likelihood with which children identified the people with child-specific knowledge *as children* on the parents’ belief measure, country, age, and their interactions. (Note the change in the coding of the dependent variable from the identification task to facilitate interpretation of the results.) The regression analysis showed an interaction effect between parents’ beliefs and country, β = .92, *p* = .018, and no other effects (see Table 2). The partial correlation between parental beliefs and the likelihood with which Canadian children identified the characters as children, controlling for age, was not significant .15, *p* = .3. For Japanese children, this correlation was -.35, *p* = .029. Following the negative sign of the correlation, the more likely caregivers were to profess a belief that their children sometimes knew more then they knew, the less likely their children were to identify the characters possessing child-domain knowledge as children.

**Table 2:** Relation between Parental and Child Beliefs.

Variable	*β*	*t*	*p*	*F*	*df*	*p*	*adj*.*R*^*2*^
**Overall model**				9.713	7, 80	.000	.59
Parental beliefs	-.501	-1.676	.098				
Country (1 = Canada)	-.362	-.951	.344				
Grade (1 = 2^nd^ grade)	.441	1.012	.315				
Country * Grade	.234	.542	.589				
Country* Parental Beliefs	.922	2.408	.018				
Grade *Parental Beliefs	.347	.754	.453				
Country* Grade *Parental Beliefs	-.679	-1.554	.124				
**Canada Model**				6.3	2, 44	.004	.19
Parental beliefs	.146	1.017	.315				
Grade (1 = 2^nd^ grade)	.397	2.761	.008				
**Japan Model**				29.679	2, 38	.000	.59
Parental beliefs	-.230	-2.264	.029				
Grade (1 = 2^nd^ grade)	.750	7.398	.000				

*Note*: The dependent variable is the proportion of time the characters of the child knowledge items were identified as children.

## Discussion

The goal of the present research was to examine the development of beliefs about adult- and child-specific knowledge. Replicating previous findings with North American children [33–35] and extending them to another culture, our findings reveal remarkable similarity between Canadian and Japanese children. Four-year-olds in both countries displayed beliefs about existence of adult-specific knowledge. They also discriminated between child and adult knowledge items, demonstrating recognition that different pieces of information are not equally familiar to different subsets of people. However, neither Canadian nor Japanese 4-year-olds showed awareness that children may know more than adults. In contrast, 7-year-olds displayed robust beliefs that both adults and children are more likely to know certain things than the other group. Across cultures, children appear to develop beliefs about the existence of adult-specific knowledge before beliefs about the existence of child-specific knowledge, and the latter appear to consolidate around the onset of elementary school.

This similarity in development may grow out of a biologically grounded core framework that provides a conceptual base of theory-of mind-development, such as a psychological construal of people in mentalistic terms [57]. Similarity in experience may also explain similarity in development. Certainly, Canadian and Japanese cultures provide similar relevant experience in a number of ways. Both are industrialized nations where institutions such as school serve not only to teach children various skills but mark the separation of the child and adult experience [58]. In addition, both Canadian and Japanese parents wish to help their children achieve a balance between being a unique individual and a community member [44,45,59].

Another possibility, however, is that similarity in developmental outcomes results from the work of overlapping but not identical set of factors. Despite major similarities, Canada and Japan present strikingly different world views which are reflected in the organization of child rearing practices and the structuring of child-peer interactions [17,51]. Our findings are consistent with such equifinality in development and proposals for diverse pathways in socio-cognitive development across cultures [14,17,18,52,60]. Canadian children’s self-reported knowledge was positively correlated with their identification decisions. The effect, however, was relatively weak as it disappeared when the items were split by domain. In fact, neither Canadian nor Japanese children’s self-reported knowledge were significantly related to their responses to adult-domain items. There was a difference, however, on child-domain items, with Japanese but not Canadian children showing a relation between self-reported knowledge and identification decisions. This is in keeping with the proposal that Japanese children have stronger beliefs about similarity between self and peers emerging from the organization of peer relations. While both individuality and interdependence are important in peer settings, Japanese children are encouraged to support the group’s cohesion and engage in interdependent, coordinated activities to a greater extent than Canadian children [43,47,49].

Of note, seeing an effect of self-reported knowledge for child-domain but not adult-domain items is not surprising. Beliefs about adult-specific knowledge are established by four [33–35] and may be formed in somewhat different ways.

Another factor we examined was parental beliefs. Here, we found no evidence for a positive correlation between parent and child beliefs about child-specific knowledge in either Canada or Japan. This finding questions whether parental beliefs about child-specific knowledge play a role in the formation of corresponding beliefs in children. The negative correlation we found in Japan may suggest such a role, e.g., in the form of children rejecting parental attitudes. We find this unlikely, however, given that the parental attitudes in this case are in children's favor. Concordance between parent and child cognitions has been shown in other areas of theory-of-mind research and social cognition [36,37,61]. It is possible that the abundant, first-hand experience that children have with other children and adults makes children’s representation of the knowledge of these groups less susceptible to parental influence. Further research is needed to determine in what areas of socio-cognitive functioning there is concordance between child and adult beliefs and why differences may exist.

The findings concerning the relation between parent and child beliefs about child-specific knowledge are nevertheless consistent with predictions stemming from considerations of the embodiment of independence and interdependence values in children’s environment [17,18]. The greater emphasis on anticipating the needs of the child in Japanese family settings [50,51,53,62] creates an environment with opposing effects on parent and child beliefs: while increasing parental beliefs about child-specific knowledge, it decreases children’s willingness to attribute knowledge exclusively to children. In contrast, the emphasis on personal space, independence of movement and verbal expression in North American family settings create an environment where parent and child attention, and consequently beliefs, are disconnected. Further research is needed to fully explore these findings and the potential role of parenting practices.

The examination of the parental responses to the questions about child-specific knowledge showed that Japanese parents were more likely to endorse child-specific knowledge than Canadian parents. This ancillary finding is consistent with findings that Japanese adults of all ages are more likely to recognize the existence of different perspectives and the limits of their own knowledge than American adults [54]. Here, we extend these previous findings to attitudes concerning children. One can ask, however, given the form of the questions in our study, whether the difference in answers reflects a greater yes-bias in Japanese parents. Further analyses, described in S3 Appendix, suggested that this is unlikely.

The effects of children’s self-reported knowledge and parental beliefs were not qualified by age. This is intriguing, as it could be expected that age-related changes in the influence of these factors relate to the changes in children’s beliefs about child-specific knowledge. Instead, our data suggest developmentally stable effects of these factors in the two cultures. This is consistent with proposals for gradual development of beliefs about child-specific knowledge [33]. Furthermore, even though children’s skills and relationships change between ages 4 and 7, the cultural values of independence and interdependence continuously shape their environment.

In sum, despite the similarities in developmental outcomes, Canadian children’s beliefs about child-specific knowledge appear to function independently from their beliefs about their own knowledge while Japanese children’ beliefs about child-specific knowledge appear to be strongly related to them. Neither Canadian nor Japanese children’s beliefs about child-specific knowledge were positively correlated with parental beliefs but we found a negative correlation between child and parent beliefs in Japan. While parental cognitions may not directly influence children’s beliefs, in Japan the two appear to be interdependent, possibly as a result of the organization of parent-child relationship.

### Implications

Our results have implications for at least three areas of research: cultural learning, social category representation, and theory of mind development. First, cultural learning involves vertical (e.g., from parents to children) and horizontal (within an age cohort) transmission of knowledge [63]. Research across cultures has demonstrated that while vertical transmission dominates early childhood, horizontal transmission and the influence of peers become more important in later development [64–66]. One question this literature raises is whether the influence of adults reflects children following an age-based heuristic or adaptive decision-making based on beliefs about competence. Wood et al. [31], for instance, found that while the age of a person significantly influenced 5-year-olds’ tendency to copy causally irrelevant actions, the person’s self-reported knowledgeability did not, suggesting the application of a heuristic. Given children’s early-developing and broad conception of adult knowledge, our research invites the question of whether the effect of age derives from children seeing adults, as a group, as more competent than children in the particular task (retrieving a sticker from a novel object). If so, then it is premature to conclude that they follow a heuristic. Indeed, children show sensitivity to individual competence in familiar domains, e.g., language, despite baseline preferences for adults [29], and informant selection decisions closely mirror beliefs about the relative knowledge of children and adults in these domains [33].

Another question is when and how horizontal transmission grows. Along with other influences such as affiliative pressures, the development of beliefs about child-specific knowledge may be part of the answer. Recognizing peers as repositories of unique information motivates epistemic openness [67].

A second implication of the present findings is that we may see cultural differences in the construction of children’s beliefs about the knowledge of other social groups. In addition to age, gender and race are stereotypically associated with differences in knowledge, and occupational categories are defined by expertise. As the development of these concepts continues to be explored [68], it is important to keep in mind the possibility raised by the present study of different correlates of development in different cultures. For example, children growing up in collectivist cultures, such as Japan, may be more likely than children growing up in North America to use their knowledge to constraint beliefs about same gender and same race individuals.

Third, while the suggestion for existence of different pathways in theory-of-mind development has been previously made [14,18,69], most of the work has been focused on false belief understanding and when it is achieved [13,70]. Relatively few empirical studies have pursued the possibility of developmental equifinality [71]. More focused explorations of potential equifinality in this and other areas of theory-of-mind development are needed. Together with research suggesting that different endpoints of development are not necessarily achieved through different mechanisms [72], findings such as ours indicate that mapping the trajectories of developmental achievements across culture may not be sufficient for drawing conclusions about the cultural universality or specificity of the underlying processes [18,19,73].

A more practical implication is that the characters children are asked to reason about may matter. In particular, the majority of studies on Japanese children’s false belief understanding show a relative delay [74–76]. Yet traditionally, the protagonists in false belief tasks are children (e.g., Sally in the unexpected transfer task). If, as the present findings suggest, Japanese children are more likely than their Western counterparts to attribute their own beliefs to peers, this design feature would lead them more often to the wrong response. Hence, it may contribute to the observed delay.

### Limitations

Further research is needed to evaluate whether Japanese children are delayed relative to Canadian children in developing beliefs about child-specific knowledge. While suggested by the present data, intermediate age groups have to be tested to exactly determine when children begin to respond above chance. Two aspects of the study methodology also have to be considered. First, despite our extensive methodological precautions, it is possible that our data provide a conservative estimate of Japanese children’s beliefs about child-specific knowledge due to the use of the honorific “-san.” Honorifics may reduce the need to rely on information about what people know to determine their age. It should be noted though that the contribution of honorifics, which are routinely used in Japanese, could be multidirectional and remains to be clarified. For example, counteracting the effect just suggested, honorifics may help construct more robust representations of age-defined knowledge domains. Second, Japanese and Canadian children may respond differently in forced-choice settings. In particular, Canadian 4-year-olds may choose randomly to indicate that both children and adults know (as suggested by Study 1 in [33]), but Japanese 4-year-olds may choose “adult.”

Further research is also needed to fully understand the formation of beliefs about age-related knowledge. With respect to the variables in the present study, we measured children’s own knowledge through self-reports. We did this because we reasoned that children’s knowledge influences their beliefs about others’ knowledge through their beliefs about their knowledge rather than directly. However, a number of studies suggest that children’s beliefs about what they know, how well they know it, and when they have learned it diverge from what they actually know, how well they know it, and when they have learned it [21,77,78]. Thus, it is an open question whether children’s knowledge itself relates to their reasoning about others’ knowledge.

A potential limitation of our materials is that the child-domain items were selected based on adult judgments. Given that less than half the children claimed knowledge of these items, it is possible to question their validity. However, as noted above, children’s self-reported knowledge does not necessarily correspond to children's actual knowledge. Even if we assume such correspondence, we also need to know how common knowledge of the items is among adults. As a reminder, child-specific knowledge is knowledge that is more typical of children than adults. Children’s self reports do not provide information about the difference of knowledge between these groups as the question posed to the children was different. Using adult experts to generate our experimental materials was the only viable approach for us given that we did not want to restrict the range of items in the study. This is generally a reliable method when used with care. Nevertheless, a more objective way of establishing child- (and adult-)specific knowledge items, although perhaps limiting research to knowledge that is easy to verify, may usefully supplement the current approach.

Furthermore, we did not measure the cultural differences suggested in previous scholarship that we argued may shape children’s beliefs about child-specific knowledge (e.g., relative importance of respect for elderly, independence vs. interdependence, focus on empathy, etc.). The reasons for cultural differences on a variable (e.g., independence-interdependence) are not necessarily the same as the reasons for individual differences on the same variable [46]. The demographic data on our participants are in line with what we would expect for representative samples from Canada and Japan. Measuring and exploring individual variation on relevant variables, however, would be useful for understanding the relation among the macro (culture) and micro systems (family, peers) and cognitive outcomes.

### Conclusion

In closing, the present study contributes to understanding children’s age-related representations of knowledge. It shows that across cultures beliefs about the existence of child-specific knowledge are firmly in place at the onset of elementary school while beliefs about the existence of adult-specific knowledge are evident in preschool. The study also highlights the possibility that cultures may promote different development pathways to substantially similar developmental outcomes.

## Supporting Information

S1 Appendix Items Used in the Identification Task. (DOCX)

S2 Appendix Metacognitive Task. (DOCX)

S3 Appendix Supplemental Analyses of Parental Data. (DOCX)
