# Supplementary material for: Japanese and Canadian Children’s Beliefs about Child and Adult Knowledge: A Case for Developmental Equifinality?
Source: PLoS One. 2016 Sep 15;11(9):e0163018. doi: 10.1371/journal.pone.0163018 (PMC5025181; doi:10.1371/journal.pone.0163018)
Supplement: S1 Appendix — (DOCX) [file pone.0163018.s001.docx]

**S1 Appendix**

***Items Used in the Identification Task***

| Knowledge Domain | Canada | Japan |
| --- | --- | --- |
| Child Knowledge | | |
| TV Show | Courtney knows the name of Sponge Bob Squarepants’ best friend in the TV show, "Sponge Bob Squarepants".  Megan knows how to stop Swiper from stealing Dora the Explorer's backpack. | Sayuri-san knows the name of the mouse which comes out in Pitagoraswitch.  Ayumi-san knows how to do Arugorizumu-Koushin. |
| Game | Emily knows how many attack points Blue Eyes White Dragon from Yu-Gi-Oh! has.  Jennifer knows how to set a Monster card in the Yu-Gi-Oh! card game. | Kaori-san knows what Pikachu evolves into.  Maki-san knows how one can catch a pokemon. |
| Singing | Andrew knows the actions to the game "Stella Ella Olla".  Joshua knows how to move his hands for the song "I'm squishing up my baby bumblebee." | Akira-san knows the entire song of zui-zui zukkurobashi.  Takeshi-san knows how to do the moves to Arupus-Ichimanjaku. |
| Adult Knowledge | | |
| Cooking | Ryan knows what cheese is made of.  Anthony knows how to make mushroom soup. | Masao-san knows what tofu is made of.  Kazuo-san knows how to make miso soup. |
| Language | Katrina knows what the word "elaboration" means.  Hillary knows how to spell the word "pterodactyl". | Akiko-san knows what the word elaboration means.  Mayumi-san knows how to write orange with kanji. |
| Transportation | Justin knows how much a car costs.  Paul knows how to drive a bus. | Yoshio-san knows how much a car costs.  Kenta-san knows how to drive a bus. |
