# Supplementary material for: Japanese and Canadian Children’s Beliefs about Child and Adult Knowledge: A Case for Developmental Equifinality?
Source: PLoS One. 2016 Sep 15;11(9):e0163018. doi: 10.1371/journal.pone.0163018 (PMC5025181; doi:10.1371/journal.pone.0163018)
Supplement: S2 Appendix — (DOCX) [file pone.0163018.s002.docx]

**S2 Appendix**

Metacognitive Task

The metacognitive task was designed to be an explicit measure of children’s perception of the existence of child-specific knowledge. Children were asked two complementary questions in counterbalanced order: “Do you think that children *sometimes* know more than adults?” and “Do you think that adults *always* know more than children?”. For the analyses, children were grouped according to whether their responses to both questions showed a belief that adults always know more, that children sometimes know more (i.e., a belief in the existence of child-specific knowledge), or their responses to the two questions were inconsistent. Figure 3 shows the actual distribution of responses.

By chance, 25% of children would display a belief in child-specific knowledge. Separate chi-square tests showed that Canadian 7-year-olds deviated significantly from this predicted distribution, χ^2^(1, *N* = 24) = 14.92, *p* = .001, but Canadian 4-year-olds did not, χ^2^(1, *N* = 24) = .22, *p* = .8. There was a significant difference between the two age groups, χ^2^(1, *N* = 48) = 7.07, *p* = .017. Japanese 7-year-olds did not deviate significantly from the distribution that could be obtained by chance, χ^2^(1, *N* = 24) = .88, *p* = .4. Japanese 4-year-olds did, χ^2^(1, *N* = 24) = 5.56, *p* = .03, but only because the number of those endorsing child-specific knowledge was too low. As Figure 3 shows, a high number of Japanese 4-year-olds showed inconsistent beliefs (the majority responded “yes” to both questions). The distribution of response patterns did not vary by age, χ^2^(1, *N* = 48) = 2, *p* = .34. A Generalized Linear Model, including country and grade as variables, showed a main effect of grade, Wald χ^2^(1) = 8.25, *p* = .004, and country, Wald χ^2^(1) = 10.44, *p* = .001.

a)

b)

Fig 3. Proportion of children who showed support for child-specific knowledge in a) Canada and b) Japan. By chance, 25% of children would assert that children sometimes know more than adults, 25% that adults always know more, and 50% would be undecided.

Canadian children’s responses to the metacognitive questions were consistent with the results from the identification task, suggesting that whereas 7-year-olds are willing to explicitly assert the existence of child-specific knowledge, 4-year-olds are not. Controlling for the effect of age (in months), there was a significant positive correlation between their scores on the two tasks (*r* = .415, *p* = .004).

The high rates of inconsistency of the Japanese children in the metacognitive task suggests that the task was likely inappropriate to capture their beliefs. Not surprisingly, their performance on this task and the identification task were not correlated (*r* = .03).
