# Supplementary material for: Japanese and Canadian Children’s Beliefs about Child and Adult Knowledge: A Case for Developmental Equifinality?
Source: PLoS One. 2016 Sep 15;11(9):e0163018. doi: 10.1371/journal.pone.0163018 (PMC5025181; doi:10.1371/journal.pone.0163018)
Supplement: S3 Appendix — (DOCX) [file pone.0163018.s003.docx]

# S3 Appendix

Are Japanese parents more likely than Canadian parents to say that their children know things that they do not due to a yes-bias?

If so, Japanese parents may be expected to provide as many or fewer examples of child-specific knowledge as Canadian parents. However, this was not the case. The median number of examples of child-specific knowledge (averaged for the two questions) provided by Japanese parents who had responded affirmatively to the questions was 3 for both 4-year-olds’ parents (range 1 – 11) and 7-year-olds’ parents (range 1 – 5.5). In contrast, the median number of examples provided by Canadian parents who responded affirmatively was 1 for 4-year-olds’ parents (range 1 – 3.5) and 1.75 for 7-year-olds’ parents (range 1 – 3). Thus, Japanese parents were both more likely to say that their children knew things that they did not and to provide more examples to support their answers. Of note, to other questions in the questionnaire which required enumeration (e.g., What do you and your child talk about together?), Canadian parents provided slightly more examples than Japanese parents. Thus, the greater number of examples provided by Japanese parents to the child-specific knowledge questions is unlikely to be due to general loquaciousness.
